# Determining optimal diagnostic criteria through chronicity and comorbidity

**DOI:** 10.1186/s40203-016-0015-8

**Published:** 2016-02-01

**Authors:** Douglas Steinley, Sean P. Lane, Kenneth J. Sher

**Affiliations:** University of Missouri and the Midwest Alcoholism Research Center, Columbia, MO USA; Department of Psychological Sciences, University of Missouri-Columbia, 210 McAlester Hall, Columbia, MO 65203 USA

**Keywords:** Alcohol use disorder, Chronicity, Comorbidity, DSM, Optimization

## Abstract

**Purpose:**

Contemporary approaches to clinical diagnosis have not adequately exploited state-of-the-art empirical techniques in deriving diagnostic criterion sets that are statistically optimal based on 1) relevant external indicators and 2) replicability across data sets. We provide a proof of concept that optimal criterion sets can be derived with respect to alcohol use disorder (AUD) diagnosis that are both more efficient and precise than current systems.

**Methods:**

Using data from the National Epidemiologic Survey on Alcohol and Related Conditions we selected chronicity (i.e. persistence) of AUD diagnosis and comorbidity of AUD with other disorders as validation criteria on which to optimize the size of the AUD criterion set and the threshold for AUD diagnosis. We used cross-validation and consensus approaches for choosing a final solution.

**Results:**

Cross-validation did not produce a solution that replicated across random subsamples or differed from conventional diagnosis. Alternatively, consensus produced a more global solution that was associated with greater validity than “conventional” diagnosis.

**Conclusion:**

Such methods, if applied to extant diagnostic criteria and algorithms can generate simpler and more reliable rules and hold promise for greatly reducing misclassification of individuals in both research and applied clinical contexts.

## Background

Much of the foundational structure of clinical practice, psychiatric epidemiology, etiological research in psychopathology, and other research enterprises related to mental illness is based on psychiatric diagnosis. Progress in all of these areas requires a valid structure for classifying and quantifying psychiatric symptomatology. The modern diagnostic approach, first formalized by Kraepelin (1883), argued that distinct disorders could be characterized by careful syndromal observation coupled with course (i.e., diagnosis by prognosis). Approaches to determining how psychiatric syndromes should be carved out from the universe of symptoms have not advanced greatly since the modern diagnostic era began. Although there have been a number of important discoveries in the area of psychopathology, the arguably slow progress of scientific research in psychopathology compared to many other health-related conditions is almost certainly attributable, in part, to limitations of the existing psychiatric nosology, currently exemplified, but certainly not limited to, the Diagnostic and Statistical Manual of Mental Disorders, fourth edition (DSM-IV; American Psychiatric Association 2000) and fifth edition (DSM-5; American Psychiatric Association 2013).

As a result, traditional diagnostic approaches have been criticized in recent years, and alternatives focusing on more transdiagnostic, process-based constructs that represent specific underlying mechanisms of mental disorders in general, such as the Research Domain Criteria (RDoC; e.g. Insel et al., 2010), and addictive disorders specifically (Litten et al., 2015), have been proposed. We are sympathetic to the RDoC approach but believe that there are numerous methodological and conceptual challenges (e.g. Miller & Rockstroh, 2013) and that the field is far from implementation in the clinic on even some of the most “tried and true” RDoC measures (see Sher, 2015). Therefore, we believe that improved clinical diagnosis using extant frameworks will play an important role in advancing the field for the foreseeable future. Furthermore, the types of optimization analyses that we propose have considerable promise for parallel application within an RDoC framework, in which diagnostic criteria are replaced with transdiagnostic psychological dimensions.

In the current paper, we adopt a novel approach, enabled by recent developments in quantitative and behavioral sciences, to *empirically derive* new optimal diagnostic criteria sets and algorithms for alcohol use disorder (AUD). This research is motivated by the fact that contemporary approaches to diagnosis have not adequately exploited empirical techniques to derive criteria that could be considered optimal with respect to predicting relevant external criteria robustly and showing generalizability across data sets and populations. Indeed, the predominant approach for developing diagnostic criteria sets has been to convene expert panels to draft criteria sets informed by systematic literature reviews, with additional but limited reanalysis in some instances, and then to characterize the performance of these criteria sets with respect to traditional psychometric properties (e.g., factor structure and test-retest stability; Hasin et al., 2012) and clinical considerations (e.g., ease of use, estimated prevalences; see Hasin et al., 2013).

It is critical to note that DSM-5 and its predecessors (American Psychiatric Association, 1980, 2000, 2013), as well as the International Classification of Diseases and Related Health Problems (ICD-10) and its precursors (World Health Organization, 1977, 1978, 1992) were developed primarily for clinical practice and public health efforts. The research community was not a primary constituency in the development of diagnostic criteria. Indeed, both DSM and ICD systems of classification were developed emphasizing clinical utility over research utility. Additionally, both the APA and WHO have stressed that future iterations of these diagnostic systems should be implementable and enhance usability for the clinicians for whom they were designed (Kendler et al., 2009; Reed, 2010). Empirically derived optimal criterion sets using the existing classification systems have the very real potential to reduce the number of criteria clinicians need to consider when diagnosing clients, significantly reducing the burden of diagnosis on clinicians while at the same time increasing diagnostic validity.

Furthermore, although the DSM and ICD were developed primarily to serve clinical efforts, these systems have become the standard for making diagnoses across many, if not most, psychiatric disorders in research contexts. This is unfortunate because diagnoses derived from the DSM/ICD have never been shown to be optimal for characterizing conditions of interest. The cost of having criteria sets that are suboptimal could be immense and result in increased rates of both Type 1 (i.e., “false discoveries”) and Type 2 errors (i.e., false negative findings) in research contexts and, similarly, suboptimal diagnosis in clinical settings.

We seek to provide a proof of concept that optimal criterion sets can be derived, based on a small set of user-specified (or otherwise agreed-upon) assumptions, for AUD diagnosis. We chose AUD among the existing predefined set of disorders for a number of reasons. First it belongs to the broader group of substance use disorders (SUDs), all of which make use of a relatively simple count of endorsed criteria to determine diagnosis. This is in contrast to other disorders which sometimes structure criteria sets hierarchically (e.g. depressive and bipolar I disorders; c.f. Lane & Sher, 2014). Thus, SUDs constitute a relatively simple and concrete structure for diagnosis that can be leveraged as a first test or whether criterion set optimization can be realistically operationalized. Second, as described below, there are a number of reasons to question the accuracy and efficiency of AUD diagnosis and its criterion set.

For example, test-retest reliability for AUD in field trials of the purportedly improved DSM-5 (over DSM-IV) has not been impressive (Regier et al., 2013; Freedman et al., 2013), though design issues may be partially responsible. However, unreliability may also come from findings that indicate that some criteria are mild or normative (Tolerance), not necessarily indicative of pathology (Hazardous Use), or easily misunderstood (Larger/Longer; see Martin et al., 2011a). As a result, some researchers have suggested that the 2-of-11 rule, in reference to the number of criteria that need to be endorsed in order to qualify for diagnosis, will diagnose many whose substance involvement has questionable clinical significance (Martin et al., 2011b). Furthermore, there has been evidence suggesting that the 2/11 rule results in substantial overlap in presumed latent severity between those who are subsyndromal (i.e., 1/11 criteria met) and those at diagnostic threshold (Lane & Sher, 2014). This overlap is also observed at higher levels of syndromal severity and is suggestive of nonadditivity of the criteria when endorsed in different combinations, which could be due to predictive redundancy across criteria or because some criteria are particularly fallible by themselves but are internally validated by the presence of some specific, additional criteria (Lane & Sher, 2014).

In the following example we use data from a large nationally representative sample of United States residents to demonstrate the feasibility and utility of extracting optimal criterion sets for diagnosing AUD. We use (1) chronicity of an AUD diagnosis from baseline to follow-up and (2) comorbidity of other Axis I and Axis II disorders (also assessed at baseline) with an AUD diagnosis, as objective criteria with which to optimize diagnosis. Chronicity was chosen as one primary consideration given its recognized importance since the time of Kraepelin (see also Spitzer, 1983). Comorbidity (co-occurrence) with other disorders (Grant et al., 2005, 2006) was chosen as many psychometric studies have demonstrated that these disorders are associated with not only externalizing psychopathology (Krueger et al., 2007) but overall psychopathology more generally (Caspi et al., 2014), and it is consistent with the notion of a condition being associated with harm (Spitzer, 1999; Wakefield, 1992). We note that there are a number of alternative criteria (e.g., level of consumption, physical health, general psychosocial functioning) that could be used for optimization, but contend that these two domains provide a clinically meaningful set of criteria in that they leverage both chronicity and clinically meaningful psychopathology to identify symptom configurations that are likely to especially problematic for the individual.

## Method

### Sample

The National Epidemiological Survey on Alcoholism and Related Conditions (NESARC; Grant et al., 2003b; Grant et al., 2005) is a nationally representative sample of non-institutionalized United States civilians 18 years and older. The survey oversampled minority ethnicities (Blacks and Hispanics) and young adults between the ages of 18 and 24. The initial wave, which was administered using face-to-face interviews between 2001 and 2002 contained 43,093 respondents (Grant et al., 2003b). A second follow-up wave of assessment was conducted during 2004-2005 and contained 34,653 of the same respondents (Grant & Kaplan, 2005). The instruments used to assess disorders in both waves of the NESARC followed DSM-IV American Psychiatric Association (2000) specifications. Though Wave 2 did include criterion items assessing craving, which has since been added to the DSM-5 AUD criterion set (American Psychiatric Association 2013), its absence at Wave 1 precluded a precise estimate of DSM-5 AUD chronicity. Given that chronicity is a central validity indicator in the current study, we instead used the 11 DSM-IV AUD criteria (10 of which overlap with DSM-5), since they were fully assessed at both waves.

In the current analyses we limit our sample to the 15,773 individuals (male = 7,428 [47 %], female = 8,345 [53 %]) in Waves 1 and 2 who had consumed at least one alcoholic beverage in the past year and also did not exhibit missing data on any of the items used in the optimization procedure (e.g., all of the AUD criteria and the Axis I and Axis II comorbidity items). We did so because individuals who abstained from drinking would contribute little to no variance to both AUD criteria endorsement and the drinking-related validity measures. Individuals ranged in age from 20 to 90 years (*M* = 44.2, *SD* = 15.7), with a majority White (65.8 %) and the remainder Hispanic (16.0 %), Black (14.7 %), Asian (2.0 %), and Native American (1.5 %). Table 1 shows more demographic information for the current sample.

**Table 1:** Demographic information of respondents at Wave 1

Characteristic	N	%
Sex		
Male	7,428	47.1
Female	8,345	52.9
Race or ethnicity		
White	10,378	65.8
Black	2,315	14.7
Native American	240	1.5
Asian or Pacific Islander	316	2.0
Hispanic	2,527	16.0
Age, years		
18-29	2,969	18.8
30-44	5,666	35.9
45-64	5,170	32.8
≥ 65	1,971	12.5
Marital status		
Married or cohabiting	9,038	57.3
Widowed, separated, or divorced	3,387	21.5
Never married	3,351	21.2
Educational level		
Less than high school	1,049	9.6
High School	4,139	26.2
Some college or higher	10,125	64.2
Annual family income, $		
0-19,999	2,934	18.6
20,000-34,999	3,156	20.0
35,000-69,999	5,475	34.7
≥ 70,000	4,211	26.7
Region		
Northeast	3,302	20.9
Midwest	3,809	24.1
South	5,050	32.0
West	3,615	22.9

### Measures

#### AUD criteria

The 11 criteria used to diagnose the presence and severity of AUD include 1) increased tolerance to alcohol’s effects (Tolerance), 2) attempts to cut down or control use (Cut Down), 3) drinking larger amounts or for longer periods of time than intended (Larger/Longer), 4) giving up important social, occupational, or recreational activities (Give Up), 5) spending a lot of time obtaining, using, or recovering (Time Drinking), 6) continued use despite physical or psychological problems (Continue), 7) experiencing withdrawal symptoms (Withdrawal), 8) failure to fulfill role obligations (Home/Job), 9) using alcohol in physically hazardous situations (Hazardous Use), 10) getting arrested, held at a police station, or having other legal problems (Legal), and 11) continued use despite social and interpersonal problems (Fight/Trouble). Using DSM-IV guidelines (American Psychiatric Association 2000) for criterion endorsement each criterion was coded as present (‘1’) or absent (‘0’) in the past year based on individuals’ responses to the particular criterion items using the Alcohol Use Disorder and Associated Disabilities Interview Schedule-IV (AUDADIS-IV; Grant et al. 2003a). Using past year endorsement was critical for strictly defining chronicity in the current example such that endorsement could be mutually exclusive and assessment timeframes and associated endorsement rates would be comparable at each wave. Endorsement rates for each criterion at both waves are shown in Table 2. In addition, we also included endorsement of the craving criterion from Wave 2 as an external validation measure for comparisons between DSM-IV and our optimally derived diagnoses.

**Table 2:** DSM-IV endorsement percentages for Waves 1 and 2

Criterion	% Endorse Wave 1	% Endorse Wave 2
Larger/Longer	8.9952	11.6940
Cut Down	8.1693	10.9700
Hazardous Use	8.0196	7.8461
Tolerance	6.2062	7.5960
Withdrawal	3.0702	3.5789
Continue	3.0163	3.9440
Time Drinking	2.0648	2.1665
Fight/Trouble	1.7775	1.1970
Home/Job	.7780	.8439
Legal	.7601	.6703
Give Up	.6643	.7421

#### Axis I and Axis II diagnoses

Presence of lifetime Axis I (*AxI*) and Axis II (*AxII*) diagnoses (i.e. non-AUD/SUD) was assessed using responses from Wave 1 and Wave 2. The same eleven Axis I disorders were assessed in both the first and second waves of the NESARC; however, we focused on seven based on their high comorbidity with AUD (Major depressive episode, Dysthymia, Hypomanic episode, Panic disorder with or without agoraphobia, Social phobia, Specific phobia [though more equivocally], Generalized anxiety disorder; Sher et al., 2014). We used the lifetime diagnosis of these seven disorders as of Wave 1 as comorbidity criteria on which to derive optimal solutions and then used the same (cumulative) lifetime measures as of Wave 2 as external validation criteria on which to compare our optimal diagnostic solution with diagnoses generated by DSM-IV.

Seven Axis II disorders were assessed at Wave 1 (Avoidant, Dependent, Paranoid, Obsessive-Compulsive, Schizoid, Histrionic, and Antisocial). In contrast, only three PDs were assessed at Wave 2 (Borderline, Schizotypal, Narcissistic). For Antisocial PD, Wave 1 included the assessment of Conduct Disorder before age 15 and Adult Antisocial Behavior at or after age 15, whereas Wave 2 included only an assessment of Adult Antisocial Behavior since the last interview.

For Axis II disorders, we used lifetime diagnoses across Wave 1 and Wave 2 to facilitate aggregation given the unbalanced design. This involved using lifetime measures of the three Axis II disorders assessed at Wave 2, the lifetime measures of the six Axis II disorders assessed at Wave 1 (acknowledging the limitation that individuals could have developed these disorders after Wave 1), and an inclusive combination of individuals who qualified for either Wave 1 lifetime Antisocial PD or both Wave 2 Adult Antisocial Behavior and Wave 1 pre-age-15 Conduct Disorder. Presence/absence of each disorder was coded as present (‘1’) or absent (‘0’) and aggregated as detailed below.

#### Health indices

The ten subscale measures from the SF12-V2 Physical and Mental Functioning scales (Ware et al., 2002) were used as external validators for the optimization procedure. These included measures of physical health, mental health, a physical health subscale, role physical health, bodily pain, general health, vitality, social health, role emotional health, and a mental health subscale. Each scale was originally constructed to have a mean of 50 and a standard deviation of 10.

#### Drinking behavior

Individuals’ self-reported drinking behavior was operationalized with two survey questions. The first was a survey-derived measure of average daily volume of ethanol consumption in the last 12 months based on individuals’ reports of the quantity and frequency with which they drank. The second was the number of times an individual reported exceeding daily drinking limits (5+ drinks in a 2-h window for men, 4+ for women; Grant et al., 2003a, 2003b).

### Optimization approach

When considering how to choose a relevant diagnostic rule, persistence and two types of comorbidity were considered. Persistence is defined as the conditional probability that if a person is diagnosed at Wave 1 what is the probability he/she is diagnosed at Wave 2, given as *P(AUD2|AUD1)* in the equations presented below; naturally, 0 ≤ *P(AUD2|AUD1)* ≤ 1. The two types of comorbidity considered were the comorbidity between diagnosis of AUD at Wave 1 and a diagnosis of Axis I symptom disorders *(AxI)*, denoted as *C(AUD1, AxI)*, and the comorbidity between a diagnosis of AUD at Wave 1 and a diagnosis of Axis II personality disorders *(AxII)*, denoted as *C(AUD1, AxII)*. Here, diagnosis of AUD (i.e. *AUD1* and *AUD2*), which implicitly contains both the set of specific criteria used for diagnosis and the threshold of number of criteria required for diagnosis, is the variable being optimized, and is thus a priori, not well defined. We are agnostic to the optimal solution and so include the entire criterion set of 11 DSM-IV criteria and all possible threshold values as candidates.

The first consideration is how to appropriately measure the comorbidity between a diagnosis of AUD and an Axis I or Axis II disorder. For all variables considered, the diagnosis is a binary outcome where a “1” and “0” indicate diagnosis and no diagnosis, respectively. Two binary variables result in the possibility of four logical states: (a) a diagnosis on both variables, (b) a diagnosis on the first variable but not the second, (c) a diagnosis on the second variable but not the first, and (d) no diagnosis on either variable. For each of the four conditions, we will let *a*, *b*, *c*, and *d* represent the total number of individuals within each condition, respectively. Based on these four values several various measures of agreement can be constructed (e.g., correlation, Yule's Q, Yule's Y, odds ratio, etc.).

For the current optimization task, we utilize Jaccard's measure (1901), defined as *a/(a + b + c)*. Jaccard's measure has the advantage of ignoring *d*, the mutual absence of a trait (e.g., no diagnosis on each variable), which is necessary in situations where the overall prevalence of several variables can be comparatively small (e.g., not close to .5) as with many disorders (Grant et al., 2005, 2006; Kessler et al., 2005). Including *d* in these instances can artificially increase or decrease the similarity measure due to the high value of *d* by design. We denote *J(V1, V2)* as the agreement between variables *V1* and *V2*; clearly, 0 ≤ *J(V1, V2)* ≤ 1.

Given a choice of agreement measure, the question becomes how to measure the agreement between a single diagnosis of AUD and a construct, such as Axis I symptom disorders, which contains several individual variables. Comorbidity between AUD at Wave 1 and Axis I symptom disorders is defined as,

1 $$ \begin{array}{l}C\left(AUD,\ AxI\right) = med\Big[J\left(AUD1,\mathrm{Major}\ \mathrm{Depression}\right),\ J\left(AUD1,\mathrm{Dysthymia}\right),\\ {}\kern16em J\left(AUD1,\mathrm{Hypomanic}\right),\ J\left(AUD1,\mathrm{Panic}\ \mathrm{w}/\ \mathrm{or}\ \mathrm{w}/\mathrm{o}\ \mathrm{Agoraphobia}\right),\\ {}\kern15.5em J\left(AUD1,\mathrm{Social}\ \mathrm{Phobia}\right),\ J\left(AUD1,\mathrm{Specific}\ \mathrm{Phobia}\right),\\ {}\kern15em J\left(AUD1,\mathrm{Generalized}\ \mathrm{Anxiety}\ \mathrm{Disorder}\right)\Big]\end{array} $$

where “*med*” denotes the median. Similarly, comorbidity between *AUD1* and Axis II personality disorders is constructed from the individual comorbidities between *AUD1* and the three personality disorder clusters of Axis II; Cluster A (*CA*), Cluster B (*CB*), and Cluster C (*CC*) subgroups as

2 $$ C\left(AUD1,\  AxII\right) = .1C\left(AUD1,\ CA\right) + .7C\left(AUD1,\ CB\right) + .2C\left(AUD1,\ CC\right) $$

where,

$$ \begin{array}{l}C\left(AUD1,\ CA\right) = med\left[J\left(AUD1,\mathrm{Paranoid}\right),\ J\left(AUD1,\mathrm{Schizoid}\right),\ J\left(AUD1,\mathrm{Schizotypal}\right)\right]\\ {}C\left(AUD1,\ CB\right) = med\Big[J\left(AUD1,\mathrm{Antisocial}\right),\ J\left(AUD1,\mathrm{Borderline}\right),\ J\left(AUD1,\mathrm{Histrionic}\right),\\ {}\kern16.5em J\left(AUD1,\mathrm{Narcisstic}\right)\Big]\\ {}C\left(AUD1,\ CC\right) = med\Big[J\left(AUD1,\mathrm{Avoidant}\right),\ J\left(AUD1,\mathrm{Dependent}\right),\\ {}\kern16.5em J\left(AUD1,\mathrm{Obsessive}-\mathrm{Compulsive}\right)\Big]\end{array} $$

The general weighting scheme was based on the observation that so-called Cluster B personality disorders (esp. Antisocial and Borderline) are likely to be more highly comorbidity than Cluster A (Odd/eccentric) and Cluster C (anxious/fearful) but with a tendency for Cluster C to be slightly more associated with AUD than Cluster A and that this general ordering is consistent with the larger personality literature on AUD correlates (Sher et al. 2005; Sher et al., 1999). The general approach is to change the rule (e.g., algorithm) which determines diagnosis of AUD and compute *C(AUD1,AxI)*, *C(AUD1,AxII)*, and *P(AUD2|AUD1)* for each of the different scenarios. For the eleven criteria that are included in the DSM-IV alcohol dependence and abuse diagnoses, there are 2^11^ - 1 = 2,047 different subsets of interest (note that the empty subset is excluded). Within each subset, the total number of criteria required to be endorsed can also be varied from one up to the total number of items in the subset. For instance, within DSM-IV three out of seven of the dependence criteria are required to be diagnosed with alcohol dependence. However, the threshold of three could easily be lowered to two or raised to four. In DSM-5 American Psychiatric Association (2013), which defines AUD as a unitary construct (jettisoning the abuse/dependence distinction in DSM-IV), the diagnostic threshold is two of eleven criterion but alternative thresholds (1/11, 2/11, …11/11) could be considered. The present investigation looks at all possible thresholds for each of the criterion sets varying in size from 1 to 11, the total number of which is provided in Table 3. A flowchart of the steps in the optimization procedure is shown in Fig. 1.

**Table 3:** Number of criterion sets by set size and threshold

	# Subsets by Threshold											
# Criteria	1	2	3	4	5	6	7	8	9	10	11	Total
1	11											11
2	55	55										110
3	165	165	165									495
4	330	330	330	330								1,320
5	462	462	462	462	462							2,310
6	462	462	462	462	462	462						2,772
7	330	330	330	330	330	330	330					2,310
8	165	165	165	165	165	165	165	165				1,320
9	55	55	55	55	55	55	55	55	55			495
10	11	11	11	11	11	11	11	11	11	11		110
11	1	1	1	1	1	1	1	1	1	1	1	11
Total	2,047	2,036	1,981	1,816	1,486	1,024	562	232	67	12	1	11,264

**Fig. 1:**
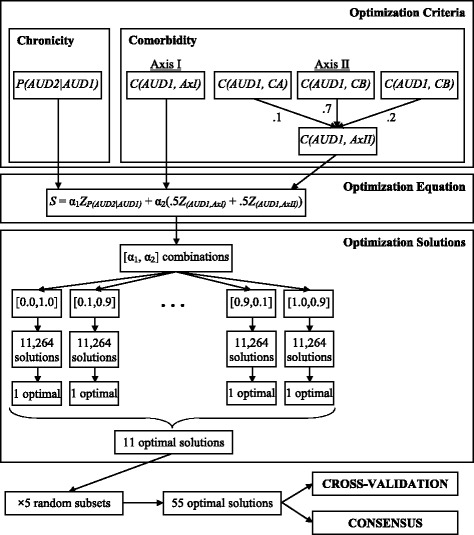
Flowchart of optimization procedure

## Results

For each of the 11,264 subsets seen in Table 3, *C(AUD1, AxI)*, *C(AUD1, AxII)*, and *P(AUD2|AUD1)* are ranked from smallest to largest and a rank-based inverse normal transformation is performed (e.g., a percentile based on the ranks is computed and the corresponding *z*-score is computed), resulting in *Z*_*(AUD1,AxI)*_, *Z*_*(AUD1,AxII)*_, and *Z*_*P(AUD2|AUD1)*_, respectively. Each of the subsets is then scored based on the formula

3 $$ S = {\alpha}_1{Z}_{P\left(AUD2\Big|AUD1\right)} + {\alpha}_2\left(.5{Z}_{\left(AUD1,AxI\right)} + .5{Z}_{\left(AUD1, AxII\right)}\right) $$

where α1 + α_2_ = 1. Furthermore, α_1_ (and by the requirement that α_1_ and α_2_ sum to one, α_2_ as well) was varied from 0 to 1 in steps of .10. The criterion set and threshold that resulted in the largest value of *S* is chosen as the optimal decision rule for the specific level of α.

### Internal validation

Given that all possible subsets are investigated, the current optimization procedure had the same faults from which most optimization procedures suffer. Namely, the results are extremely susceptible to capitalization on chance variation. To protect against this, a type of *k*-fold cross validation was conducted. The original dataset of *n* = 15,773 was randomly divided into five smaller data sets (*D*_*1*_, *D*_*2*_, *D*_*3*_, *D*_*4*_, and *D*_*5*_) with sample sizes *n*_*1*_ = 3,154, *n*_*2*_ = 3,154, *n*_*3*_ = 3,155, *n*_*4*_ = 3,155, and *n*_*5*_ = 3,155, respectively. The optimization procedure was conducted on each of the five data sets at each of the eleven α combinations, resulting in 55 potential solutions; results are provided in Table 4.

**Table 4:** Optimal rules across replication data sets

Data	α_1_	α_2_	*P(AD1)*	*P(AD2|AD1)*	*T*	1	2	3	4	5	6	7	8	9	10	11
*D*_1_	DSM-IV	DSM-IV	.042	.386	3	x	x	x	x	x	x	x				
*D*_2_	DSM-IV	DSM-IV	.041	.453	3	x	x	x	x	x	x	x				
*D*_3_	DSM-IV	DSM-IV	.039	.367	3	x	x	x	x	x	x	x				
*D*_4_	DSM-IV	DSM-IV	.042	.376	3	x	x	x	x	x	x	x				
*D*_5_	DSM-IV	DSM-IV	.031	.430	3	x	x	x	x	x	x	x				
*D*_1_	1.0 – .7	.0 – .3	.074	.464	2	x	x	x		x		x				x
	.6 – .3	.4 – .7	.052	.387	3	x	x	x		x	x	x	x	x		
	.2 – .0	.8 – 1.0	.054	.379	3	x	x	x		x	x	x	x	x		x
*D*_2_	1.0 – .8	.0 – .2	.090	.519	2	x	x	x	x	x	x	x		x		x
	.7	.3	.032	.461	3	x	x		x	x	x	x	x		x	x
	.6 – .5	.4 – .5	.040	.448	2	x			x	x	x	x	x			
	.4 – .3	.6 – .7	.041	.445	2	x			x	x	x	x	x		x	
	.2 – .0	.8 – 1.0	.029	.391	3		x	x	x		x	x	x			
*D*_3_	1.0 – .6	.0 – .4	.021	.462	3		x					x	x	x		x
	.5	.5	.041	.400	2				x		x	x	x	x	x	x
	.4	.6	.058	.348	1				x			x	x			x
	.3 – .0	.7 – 1.0	.058	.339	1							x	x			x
*D*_4_	1.0 – .8	.0 – .2	.117	.473	1		x		x	x	x	x				x
	.7 – .0	.3 – 1.0	.063	.415	2		x	x		x	x	x	x			x
*D*_5_	1.0	.0	.117	.462	1	x	x			x	x		x		x	
	.9 – .5	.1 – .5	.031	.443	2	x				x	x	x	x			
	.0 – .4	.6 – 1.0	.028	.425	2	x					x	x	X		x	
Votes						.51	.67	.49	.29	.62	.75	.98	.82	.29	.15	.60

α_1_ = weight for persistence; α_2_ = weight for comorbidity

*AD* DSM-IV alcohol dependence, *T* threshold

1 = Tolerance; 2 = Cut Down; 3 = Larger/Longer; 4 = Give Up; 5 = Time Drinking; 6 = Continue; 7 = Withdrawal; 8 = Home/Job; 9 = Hazardous Use; 10 = Legal; 11 = Fight/Trouble

The interpretation of the table is as follows. The first five rows provide the current DSM-IV diagnosis for alcohol dependence as a reference. For these solutions, the α_1_ and α_2_ weights are irrelevant. *P(AUD1)* is the prevalence of the diagnosis at Wave 1, while *P(AUD2|AUD1)* is the persistence of the diagnosis. *T* refers to the threshold of the number of criteria that must be endorsed from the specific set – which is indicated by an “x” in the appropriate column – for a diagnosis to be made. For instance, the first row of *D*_*1*_ (sixth overall) is the optimal solution for the four α combinations (α_1_, α_2_) = (1.0, .0), (.9, .1), (.8, .2), (.7, .3). The prevalence of diagnosis is 7.4 %, while the persistence of diagnosing at Wave 2 conditioned on diagnosis at Wave 1 is 46.4 %. The optimal diagnosis rule at these levels of α_1_ and α_2_ is endorsing two items out of the six criterion set: Tolerance, Cutdown, Larger/Longer, TimeDrinking, Withdrawal, Fight/Trouble.

A potentially complicating issue is that while the same solution appears several times *within* a data set (for instance, the optimal solutions for *D*_*1*_ when α_1_ = 1.0, α_1_ = .9, α_1_ = .8, and α_1_ = .7 are identical), no unique solution appears in more than one data set. To choose a final solution, one has to evaluate the response patterns observed across all five data sets in a systematic fashion. Often, for predictive modeling, there are several approaches that could be used. Here, we consider two: (a) cross-validation and (b) consensus.

Cross-validation Hastie et al. (2001) proceeds by comparing the performance of each of the optimal rules found for all of the pairs of weights (α_1_, α_2_) to each of the subsets of data that were created for the cross-validation. Table 5 provides the rank, in terms of percentage) for each of the rules for every data set. For instance, for the first row of Table 5, the rule of endorsing two items out of the six item subset of Tolerance, Cut Down, Larger/Longer, Time Drinking, Withdrawal, and Fight/Trouble corresponded to the optimal rule for *D*_*1*_ when α_1_ ranged from .7 to 1 and α_2_ ranged from .3 to 0, indicating that more weight is given to persistence and less weight is given to comorbidity. This particular rule works well for *D*_*5*_ and *D*_*4*_, appearing in the top 3.4 % and 9.6 % (recall lower is better) of solutions, respectively. However, worse performance is observed for *D*_*2*_ and *D*_*3*_, with the percentiles being 24.1 % and 38.8 %, respectively. To score each of the rules that were found, we used the maximum percentile; consequently, the “score” for this rule would be 38.8 %. Then, best solutions can be found by a simple min-max procedure (see Brusco & Steinley, 2014, for a common implementation of such a rule), where the final rule chosen is the one that minimizes the maximum percentile ranking (e.g., the rule that does the *least worst* is chosen). Inspecting Table 5, we see that endorsing 2 out of 9 items (Tolerance, Cut Down, Larger/Longer, Give Up, Time Drinking, Continue, Withdrawal, Hazardous Use, Fight/Trouble) is the best rule, with a maximum of 15.8 % when applied to the four validation data sets.

**Table 5:** Performance of rules across random subsets

Rule	α_1_	α_2_	*D*_1_	*D*_2_	*D*_3_	*D*_4_	*D*_5_	Maximum percentile
*D*_1_	1.0 – .7	.0 - .3	–	24.1	38.8	9.6	3.4	38.8
	.6 – .3	.4 - .7	–	21.6	35.5	8.5	12.5	35.5
	.2 – .0	.8 – 1.0	–	20.9	31.6	10.1	11.1	31.6
*D*_2_	1.0 – .8	.0 - .2	15.8	–	14.0	9.1	4.4	15.8
	.7	.3	19.8	–	51.2	24.9	28.4	51.2
	.6 – .5	.4 - .5	31.0	–	39.6	14.1	1.6	39.6
	.4 – .3	.6 - .7	32.2	–	35.6	12.6	1.6	35.6
	.2 – .0	.8 – 1.0	32.7	–	19.5	30.7	41.0	41.0
*D*_3_	1.0 – .6	.0 - .4	28.6	38.5	–	58.6	28.3	58.6
	.5	.5	43.0	13.0	–	32.3	24.8	43.0
	.4	.6	19.7	17.7	–	10.7	17.6	19.7
	.3 – .0	.7 – 1.0	18.4	19.7	–	10.9	16.4	19.7
*D*_4_	1.0 – .8	.0 - .2	20.0	27.2	10.7	–	7.6	27.2
	.7 – .0	.3 – 1.0	9.6	13.4	25.9	–	6.8	25.9
*D*_5_	1.0	.0	29.3	32.4	13.0	14.0	–	32.4
	.9 – .5	.1 - .5	27.9	7.0	43.9	14.1	–	43.9
	.0 – .4	.6 – 1.0	39	18.8	64.3	21.2	–	64.3
DSM-IV	NA	NA	11.9	12.7	42.3	9.5	14.2	42.3
Consensus	NA	NA	18.4	11.4	15.4	8.8	25.7	25.7

Alternatively, instead of choosing a rule based on specific weightings of persistence and comorbidity, one could derive a natural “consensus” group of criteria among a given set (see Steinley, 2008, for using consensus to determine most similar observations), with each appearing in optimal solutions a minimum percentage of the time. The importance of individual criteria can be determined by the proportion of times that they appear in each of the 55 solutions, provided in the final row of Table 4 (i.e. “Votes”). The order of importance (with percent of times in the optimal solution in parentheses) is: Withdrawal (98 %), Home/Job (82 %), Continue (75 %), Cut Down (67 %), Time Drinking (62 %), Fight/Trouble (60 %), Tolerance (51 %), Larger/Longer (49 %), Give Up (29 %), Hazardous Use (29 %), and Legal (15 %). If we chose 60 % as the cutoff for consensus that is considered markedly above chance we would retain six criteria. Following the guidelines mentioned in the introduction warning against allowing single-criterion diagnosis, the obvious threshold would be endorsing two items out of the six item set [Withdrawal, Home/Job, Continue, Cut Down, Time Drinking, Fight/Trouble]. This leads to an overall solution that does not appear in any of the individual optimizations; however, it benefits from not being tied directly to a specific weighting of comorbidity and persistence, rather, it is “nominated” from the set of all solutions that were found to be optimal.

### External validation

To determine which internal validation procedure led to a solution that performed best, both were compared on a set of external variables that are thought to be correlates with alcohol diagnosis. The variables used were a set of variables assessed at Wave 2 by the SF-12-V2 health survey (Ware et al., 2002), where larger scores indicate healthier individuals. In addition to general health variables, three alcohol consumption variables from the second wave of measurement were examined (DSM-5 AUD craving, volume of ethanol consumption, and number of times an individual exceeded 4+/5+ drinks). Finally, a series of anxiety and mood disorders were also examined for significant differences (major depression, dysthymia, hypomania, panic disorder, social phobia, specific phobia, and generalized anxiety). All external validators were tested via planned comparisons that took into account sample weights to insure the results were generalizable to the broader population.

Of the two approaches, only the consensus approach identified differences on the external variables; as such, presentation of the results for the solution identified via cross-validation is omitted. Table 6 shows the 2 × 2 cross-classification table comparing those who diagnosis under the existing DSM-IV schema for alcohol dependence (3 of 7 criteria) and those who diagnose under the consensus method (2 of 6 criteria, which included both abuse and dependence criteria). Five hundred thirty-six individuals diagnose under both approaches, while 145 diagnose under consensus only and 189 diagnose under DSM-IV only.

**Table 6:** Cross-classification of DSM-IV diagnosis and consensus diagnosis

	DSM-IV – Yes	DSM-IV – No
Consensus – Yes	536 (Both)	145 (Consensus Only)
Consensus - No	189 (DSM-IV Only)	14906 (No Both)

To assess whether there were any significant differences between the individuals in these three subgroups (Consensus Only, DSM-IV Only, and Both) on the set of external validators, planned comparisons were conducted (see Table 7). Table 7 consists of three primary comparisons: Consensus versus DSM-IV; Consensus versus Both; DSM-IV versus Both. Each of these comparisons is discussed in turn.

**Table 7:** Results of external validation

Variable	Consensus only vs. DSM-IV only			Consensus only vs. both			DSM-IV only vs. both		
	*Diff.*	*t*	*p*	*Diff.*	*t*	*p*	*Diff.*	*t*	*p*
Phys. Hlth.	−.89	−.77	.44	.23	.23	.82	1.11	.97	.26
Ment. Hlth.	−2.21	−1.92	.06	.66	.57	.57	**2.86**	**2.91**	**<.01**
Phys. Health (Sub)	−.62	−.55	.58	.64	.68	.50	1.26	1.39	.17
Role Phys.	−1.57	−1.29	.20	−.64	−.61	.54	.92	.90	.37
Bodily Pain	−.66	−.41	.68	1.42	1.10	.27	2.08	1.58	.12
Gen. Hlth.	−1.87	−1.40	.17	.70	.60	.55	**2.57**	**2.17**	**.03**
Vitality	−2.58	−1.85	.07	**−2.45**	**−2.07**	**.04**	.12	.12	.90
Social	−2.10	−1.88	.06	2.38	1.94	.06	**4.49**	**4.27**	**<.01**
Role Emotional	−1.30	−1.00	.32	2.13	1.81	.08	**3.44**	**2.86**	**<.01**
Ment. Hlth. (Sub)	−1.85	−1.41	.16	-.24	-.20	.84	1.60	1.59	.12
Craving	−.03	-.66	.51	**.25**	**5.03**	**<.01**	**.28**	**6.55**	**<.01**
Ethanol Consump.	**.69**	**3.03**	**<.01**	**−1.12**	**−2.22**	**.03**	**−1.80**	**−3.81**	**<.01**
Major Depression	.03	.80	.43	−.04	−1.03	.31	−.07	−2.18	.03
Dysthymia	.03	1.45	.15	.01	.35	.73	−.03	−1.73	.09
Hypomania	.03	1.72	.09	.00	.05	.95	−.03	−2.92	<.01
Panic Disorder	.03	1.25	.21	.00	−.14	.89	−.03	−1.99	.05
Social Phobia	.00	-.06	.95	.00	.11	.91	.01	.21	.84
Specific Phobia	.06	1.48	.14	.00	−.10	.92	−.07	−2.16	.03
Gen. Anxiety	.01	.31	.76	−.03	−1.07	.28	−.03	−1.67	.10
5+ Drinks	.37	.92	.36	**2.01**	**5.65**	**<.01**	**1.65**	**5.24**	**<.01**

All validation variables were assessed at Wave 2. The validators major depression, dysthymia, hypomania, panic disorder, social phobia, specific phobia, and generalized anxiety are all from Wave 2, whereas the same variables from Wave 1 were used as part of the comorbidity measure. The bolded numbers in the table indicate significant differences

#### Consensus (only) vs. DSM-IV (only)

This comparison is comparing the individuals in the off-diagonal cells of Table 6 (e.g., the 189 DSM-IV diagnoses and the 145 Consensus diagnoses). The comparisons of the individuals who only diagnose under one of the two rules help assess whether the Consensus classification is largely redundant with the DSM-IV classification. These comparisons correspond to the first three columns in Table 7, where it is seen that there are two significant differences. Specifically, the individuals in the Consensus only diagnosis had higher amounts of ethanol consumption (*t* = 3.03, *p* < .01).

#### Consensus (only) vs. Both

This comparison is assessing whether adding those that diagnose under the Consensus only model dilute the severity of the individuals in the more extreme “Both” category. While the Consensus only group has lower levels of ethanol consumption (*t* = -2.22, *p* = .03), they experience decreased vitality (*t* = -2.45, *p* = .04), increased craving (*t* = 5.03, *p* < .01), and more instances of consuming five or more drinks (*t* = 5.65, *p* < .01).

#### DSM-IV(only) vs. Both

This comparison is assessing whether removing individuals who only diagnose under the DSM-IV scheme would reduce the severity of the individuals in the more extreme “Both” category. In this case, it is found that for many of the external validators the DSM-IV only group has better functioning, drinks less alcohol, and has fewer associated personality disorders. The only variables that exhibit more severity for the DSM-IV group are craving (*t* = 6.55, *p* < .01) and more instances of consuming five or more drinks (*t* = 5.24, *p* < .01).

## Discussion

The basic approach to clinical diagnosis has remained relatively unchanged for over 100 years. However, we present an alternative computational approach that empirically derives optimal criterion sets and thresholds for diagnosis that makes use of extant data. This approach is in line with previously proposed standards for evaluating diagnostic interviews (e.g. Spitzer, 1983) by making fuller use of a broad set of existing data, but goes a step further by also using modern computational advances to provide objective assessments of all possible combinations of diagnostic criteria based on a set of user-selected validity measures. Similarly, in line with recent criticisms regarding content overlap and super-/sub-additivity of criterion information (Lane & Sher, 2014; Martin et al., 2014) the current approach implicitly adjusts for such combinations in its pruning of criteria that do not add additional diagnostic information.

Our application of this approach to AUD using its chronicity and comorbidity with other disorders as validity measures on which to find optimal solutions produced a diagnostic criterion set and threshold limit for diagnosis that was considerably more parsimonious (6 versus 11 criteria) and efficient at classifying those with associated alcohol problems (Table 7). Reducing the size of the criterion set without loss of precision has clear utility in reducing assessment burden, both on researchers conducting empirical studies and on clinicians treating patients. Furthermore, we observed that diagnosis as determined by our optimal solution contains individuals with more severe alcohol consumption and mental and physical health problems compared to those who only diagnose with DSM-IV alcohol dependence. Using our optimal solution as the standard, these individuals may be considered falsely diagnosed “diagnostic impostors” (Langenbucher et al., 1996) under the DSM-IV algorithm given that they were diagnosed but had significantly less impairment than those diagnosed under both algorithms. This suggests that the optimal algorithm derived by our analyses would result in fewer Type 2 errors, assuming our external validation variables did represent constructs with theoretically positively overlapping content with the underlying disorder.

While this approach represents a significant advancement in determining optimal criterion sets for clinical diagnosis, the specific example we present represents only a first step and proof of concept of more tailored approaches that can be utilized. In the current example we made assumptions about the weightings of the different Axis II clusters and the equal weighting of Axis I and Axis II disorders in estimating optimal alpha parameters. We constrained these in the current example due to the exponential increase in permutations and computation time that would result in freeing them, though future extensions could consider this. Similarly, in the current application we used a binary threshold rule for diagnosis, as in DSM-IV, though the model could be expanded to incorporate a graded response scale as in DSM-5. Lastly, and perhaps most significantly from a substantive perspective, the current approach is agnostic as to the validity criteria that it is given to find an optimal solution. We selected two measures (chronicity and comorbidity) for their clinical and historical relevance. However, others may disagree and consider different measures to be more diagnostic and objective (e.g., biomarkers). The same approach can be used to identify optimal solutions with different measures and with more or fewer measures. We do find it of interest that the consensus ranking of criteria placed both hazardous use and legal problems last among candidate criteria. The problems with legal problems has been increasingly recognized in recent years and, in fact, was the only criterion from DSM-IV not retained in DSM-5. Although hazardous use was retained in DSM-5, we Martin et al. (2011a) had advocated against its inclusion in DSM-5 on conceptual grounds. That is, the approach we have employed here appears not only useful for identifying efficient criteria sets and algorithms but also for identifying potentially problematic criteria.

### Limitations

One potential limitation is that the optimized rule is drawn from a pool of 11 items, while the DSM-IV dependence criteria is drawn from a fixed pool of 7 items, allowing the greater capitalization on chance by the Optimal Approach. This potential shortcoming is mitigated by relying on the Consensus Approach to determining the final rule instead of any one analysis. Further, it highlights both the dangers of the bifurcation of items as operationalized in DSM-IV and, due to the irrelevance of some items as related to the optimization criteria. As mentioned above, future research will extend this approach to the DSM-5 criteria set (which includes craving) due to the likely fact that pooling all items into one group to obtain a resultant sum score introduces unneeded noise into the diagnostic algorithm. Indeed, we would have employed the DSM-5 criteria set in the current series of analyses had we had a sufficiently large sample assessing craving at more than one time point but, unfortunately, NESARC only assessed craving at Wave 2.

## Conclusion

We provide a worked example of how optimal sets of diagnostic criteria can be computationally derived from the existing AUD criterion set using multiple external validity measures that are broadly accepted as robust indicators of disorder. Given the increasing availability of large-scale, population-based epidemiological studies, similar procedures can be applied to criterion sets for a wide array of other disorders and can be mobilized to improve efficiency and precision within both research and healthcare domains. Such steps represent advances that a number of researchers and clinicians alike have been advocating for years and, at the very least, can complement the expert panels and literature reviews when revising diagnostic criterion sets and diagnosis thresholds.
